# Evidence That a Laminin-Like Insect Protein Mediates Early Events in the Interaction of a Phytoparasite with Its Vector's Salivary Gland

**DOI:** 10.1371/journal.pone.0048170

**Published:** 2012-10-31

**Authors:** Felipe de Almeida Dias, Andre Luis Souza dos Santos, Letícia Miranda Santos Lery, Thiago Luiz Alves e Silva, Mauricio Martins Oliveira, Paulo Mascarello Bisch, Elvira Maria Saraiva, Thaïs Cristina Souto-Padrón, Angela Hampshire Lopes

**Affiliations:** 1 Instituto de Microbiologia Paulo de Goes, UFRJ, Ilha do Fundao, Rio de Janeiro, Rio de Janeiro, Brazil; 2 Instituto de Bioquimica Medica, UFRJ, Ilha do Fundao, Rio de Janeiro, Rio de Janeiro, Brazil; 3 Instituto de Biofisica Carlos Chagas Filho, UFRJ, Ilha do Fundao, Rio de Janeiro, Rio de Janeiro, Brazil; Technion-Israel Institute of Technology, Israel

## Abstract

*Phytomonas* species are plant parasites of the family Trypanosomatidae, which are transmitted by phytophagous insects. Some *Phytomonas* species cause major agricultural damages. The hemipteran *Oncopeltus fasciatus* is natural and experimental host for several species of trypanosomatids, including *Phytomonas* spp. The invasion of the insect vectors' salivary glands is one of the most important events for the life cycle of *Phytomonas* species. In the present study, we show the binding of *Phytomonas serpens* at the external face of *O. fasciatus* salivary glands by means of scanning electron microscopy and the *in vitro* interaction of living parasites with total proteins from the salivary glands in ligand blotting assays. This binding occurs primarily through an interaction with a 130 kDa salivary gland protein. The mass spectrometry of the trypsin-digest of this protein matched 23% of human laminin-5 β3 chain precursor sequence by 16 digested peptides. A protein sequence search through the transcriptome of *O. fasciatus* embryo showed a partial sequence with 51% similarity to human laminin β3 subunit. Anti-human laminin-5 β3 chain polyclonal antibodies recognized the 130 kDa protein by immunoblotting. The association of parasites with the salivary glands was strongly inhibited by human laminin-5, by the purified 130 kDa insect protein, and by polyclonal antibodies raised against the human laminin-5 β3 chain. This is the first report demonstrating that a laminin-like molecule from the salivary gland of *O. fasciatus* acts as a receptor for *Phytomonas* binding. The results presented in this investigation are important findings that will support further studies that aim at developing new approaches to prevent the transmission of *Phytomonas* species from insects to plants and vice-versa.

## Introduction

Trypanosomatids of the genus *Phytomonas* are parasites of insects and plants. Species of the genus *Phytomonas* are found in a wide range of geographical areas, including Northern and Central Africa, China, India, several European countries, and on the American continent [1]–[4]. The parasitism may occur without any apparent pathogenicity in the plants, but may also cause devastating diseases in plantations of economic significance. These parasites live in the phloem or lactiferous ducts of the infected plants and have also been detected in fruits, such as pomegranates, peaches, guavas, and tomatoes [4], [5]. *Phytomonas serpens* is a parasite of the tomato that use *Phthia picta* (Hemiptera: Coreidade) and *Nezara viridula* (Hemiptera: Pentatomidae) as natural hosts [6]. The phytophagous insect *Oncopeltus fasciatus* is a natural host of *Phytomonas elmasiani* [7] but is also able to host other species of trypanosomatids, as determined by experimental infection [8].

In the biological cycle of *Phytomonas* species, the parasites are ingested when a phytophagous insect feeds on an infected plant, then the flagellates pass through the intestinal epithelium and reach the hemolymph. After traveling throughout the hemocele, the protozoans reach the external face of the salivary glands. Once the parasites successfully bind to the external face of the gland, they pass through the gland epithelium and infect the salivary gland lumen. When the infected insect feeds on another plant, the flagellates are then transmitted via saliva. Therefore, the interaction between plant trypanosomatids and the vectors' salivary glands is vital for parasite transmission [5], [6], [9].

The pair of trilobed salivary glands of *O. fasciatus* is composed of a layer of simple cubical epithelium mounted on a basal lamina [10]. The chemical composition of *O. fasciatus* salivary gland basal lamina remains unknown. In other insects, the composition of basal lamina of distinct tissues is heterogeneous, but the protein laminin is regularly present [11]–[16]. Laminins belong to a family of glycoproteins that are assembled as heterotrimers of α, γ and β chains [17], [18]. The presence of laminin as receptors for parasites has been reported in mammalian systems, including the trypanosomatids *Trypanosoma cruzi* [19], [20] and *Leishmania donovani* [21]. Also, laminins have been reported as receptors for parasites in invertebrate systems, playing an essential role in the interaction of malaria parasites with their insect vectors [16], [22]–[26].

Considering that plant infections caused by *Phytomonas* species can be devastating for agriculture, blocking the entrance of parasites into insect vectors' salivary glands could be viewed as a strategy for preventing the diseases they transmit. In the present study, we investigated the *ex vivo* interaction of *P. serpens* with *O. fasciatus* salivary glands by scanning electron microscopy and the *in vitro* interaction of living parasites with total proteins from the salivary glands using ligand blotting assays. We show here that the parasites bound to a 130 kDa salivary gland protein (p130), which was identified as a laminin-5 β3 chain-like protein by mass spectrometry. These results suggest that the binding of the plant trypanosomatid *P. serpens* to salivary glands of insect vectors, which is a crucial step for the life cycle of this parasite, first occurs through an interaction with a laminin β chain-like protein.

## Results

### *Ex vivo* interaction of *Phytomonas serpens* with *Oncopeltus fasciatus* salivary glands

**Figure 1:**
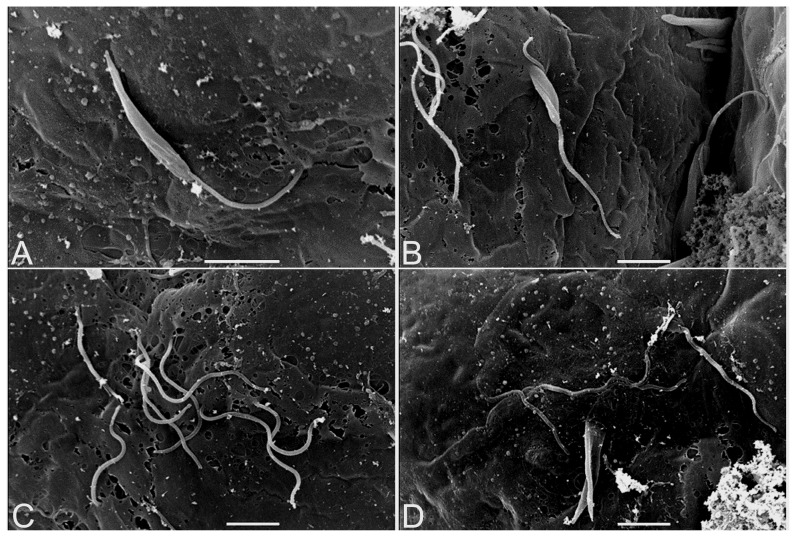
Scanning electron microscopy of *in vitro* interaction between *P. serpens* and explanted *O. fasciatus* salivary glands. The salivary glands were explanted and incubated in the presence of a suspension of parasites. (**A**) Parasite adhesion to the salivary gland basal lamina occurs through the flagellum and the cell body. (**B**) Penetration of the parasites into the salivary gland started by the parasite cell body. (**C**) Several parasites are invading the same area of the basal lamina clearly through the cell body, as their flagella can be observed outside. (**D**) Parasites after penetration covered by basal lamina. The arrows indicate suggestive lesions in the basal lamina (**B**–**D**). Flagellum (F); parasite cell body (B). Scale bars: 5 µm.

*P. serpens* parasites harvested in the stationary phase of growth were incubated in the presence of explanted salivary glands from *O. fasciatus*. Scanning electron microscopy (SEM) of the external face of the salivary glands showed a close association between the parasites and the basal lamina, where the adhesion of *P. serpens* occurred either through the flagellum or through the cellular body (Fig. 1A). On the other hand, the invasion of the basal lamina occurred only through the protozoan body (Fig. 1B), as after penetration of the parasites, some flagella were observed at the outer surface of the salivary glands (Fig. 1C). Parasites beneath the basal lamina are observed in Figure 1D. The disruption of the basal lamina during parasite-salivary gland interaction was also observed (Figures 1B and 1C).

### *In vivo* experimental infection of *Oncopeltus fasciatus* salivary glands with *Phytomonas serpens*

**Figure 2:**
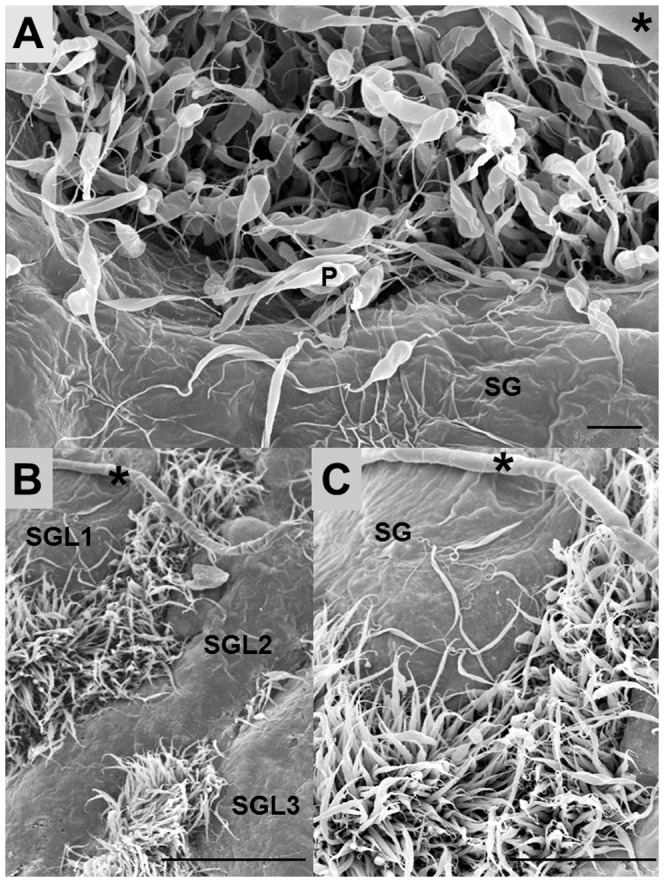
Scanning electron microscopy of *Oncopeltus fasciatus* salivary glands infected with *Phytomonas serpens*. The parasites were injected laterally into the thorax of the insects and the salivary glands were explanted at different time points after injection. (**A**) Outer surface of the salivary gland showing a high number of parasites between two salivary gland lobes and a few parasites attached to the gland, 48 h post-infection. Scale bar: 10 µm. (**B–C**) Large numbers of parasites bound to the salivary gland, 72 h post-infection. Scale bars: 100 µm (**B**) and 50 µm (**C**). SG: salivary gland; P: parasite; SGL1, SGL2 and SGL3: salivary gland lobes. The asterisk (*) indicates the salivary duct. For further details see the Methods.

Parasites were injected into the thorax of the insects. The salivary glands of the infected insects were obtained by gently pulling off their heads [27]. Parasite adhesion to the outer surface of the salivary glands was observed by SEM. Parasite binding to the salivary glands was detected as early as six hours post-injection (data not shown). The number of adhering parasites increased 48 h post-infection (Fig. 2A), reaching a high density of parasites attached to the glands 72 h post-infection (Figures 2B and 2C).

### Ligand blotting

**Figure 3:**
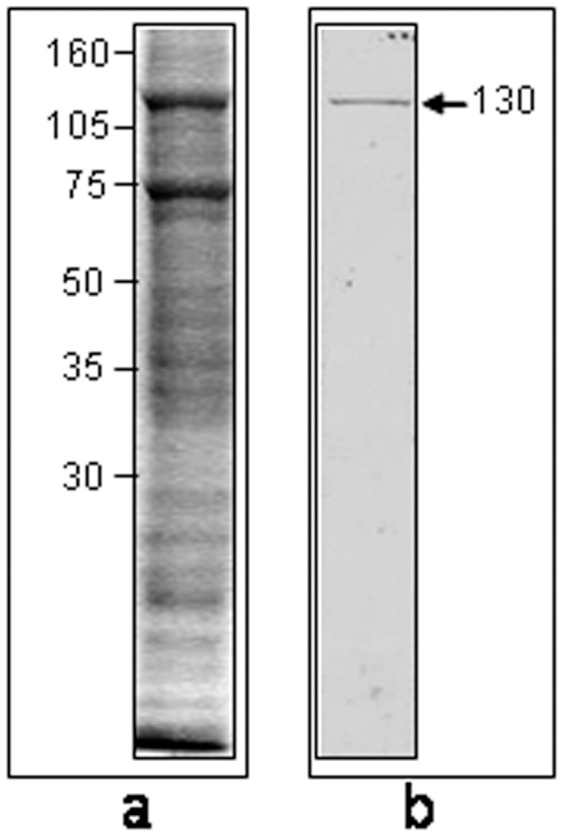
Ligand blotting showing the salivary gland proteins of *Oncopeltus fasciatus* recognized by biotin-tagged membrane proteins and live biotinylated parasites. (**Lane a**) Coomassie Blue staining of total salivary gland proteins separated by 12% SDS-PAGE. The numbers on the left indicate the relative position of molecular mass markers expressed in kilodaltons. (**Lane b**) Ligand blotting that shows the presence of a 130 kDa protein recognized by biontinylated live parasites. Prior to the incubation with the membrane, the parasite surface proteins were tagged with Sulfo-NHS-Lc-Biotin. The arrow indicates the position of the 130 kDa protein. For further details see the Methods.

In order to identify salivary gland proteins that are possibly involved in the binding of the parasites, total extract of gland proteins was transferred to polyvinylidene difluoride (PVDF) membranes (Fig. 3A). The incubation of the PVDF membranes with live parasites containing the surface proteins tagged with biotin showed that a 130 kDa salivary gland protein (p130) was recognized by these parasites (Fig. 3B).

**Figure 4:**
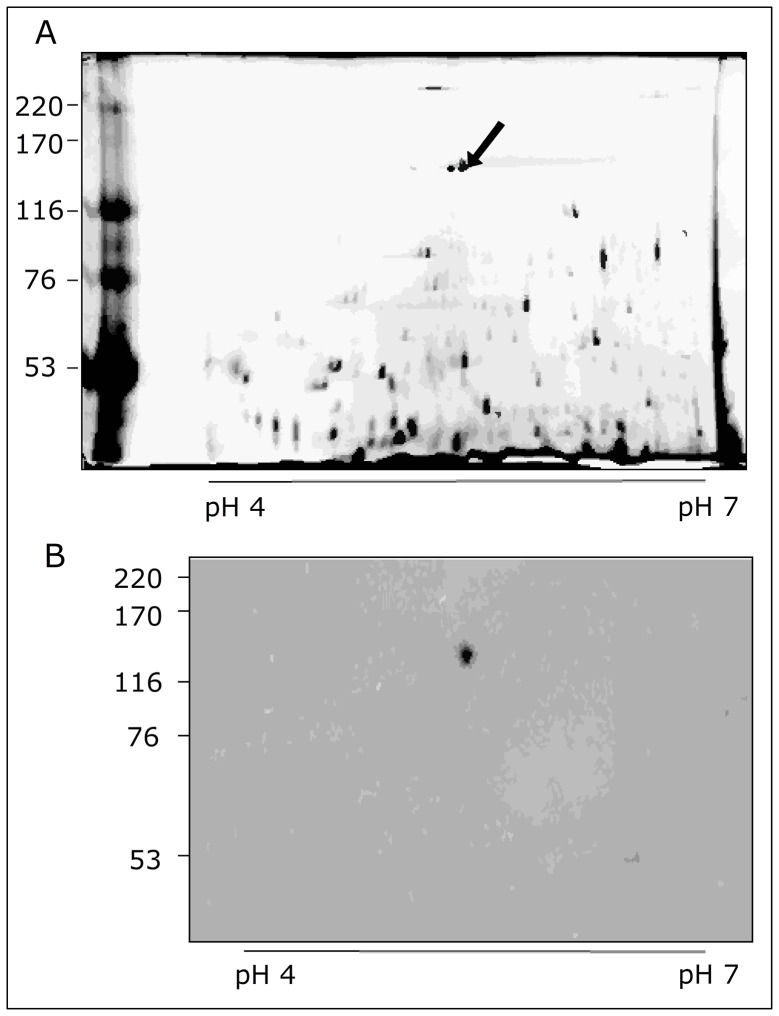
2D SDS-PAGE and corresponding ligand blotting that show the binding of live parasites to the 130 kDa polypeptide spot present in the total protein extract of *Oncopeltus faciatus* salivary glands. (**A**) 2D SDS-PAGE of total protein extract from salivary glands stained with Coomassie Blue. The arrow indicates spots at the 130 kDa region. The numbers on the horizontal axis refer to the pH gradient (pI) and the numbers on the vertical axis refer to the molecular mass standard expressed in kilodaltons. (**B**) Ligand blotting showing one spot at the 130 kDa region. The spot on the PVDF membrane indicates the binding of biotinylated live parasites to the 130 kDa protein. For further details see the Methods.

The 2D gel analysis of total salivary gland proteins showed two spots in the 130 kDa region (Fig. 4A, arrow). The polypeptides separated by 2D PAGE were then transferred to PVDF membranes. After the incubation of the membranes with biotinylated live parasites, it was observed that these parasites only bound to p130 (Fig. 4B).

### 2D PAGE analysis and mass spectrometry

**Figure 5:**
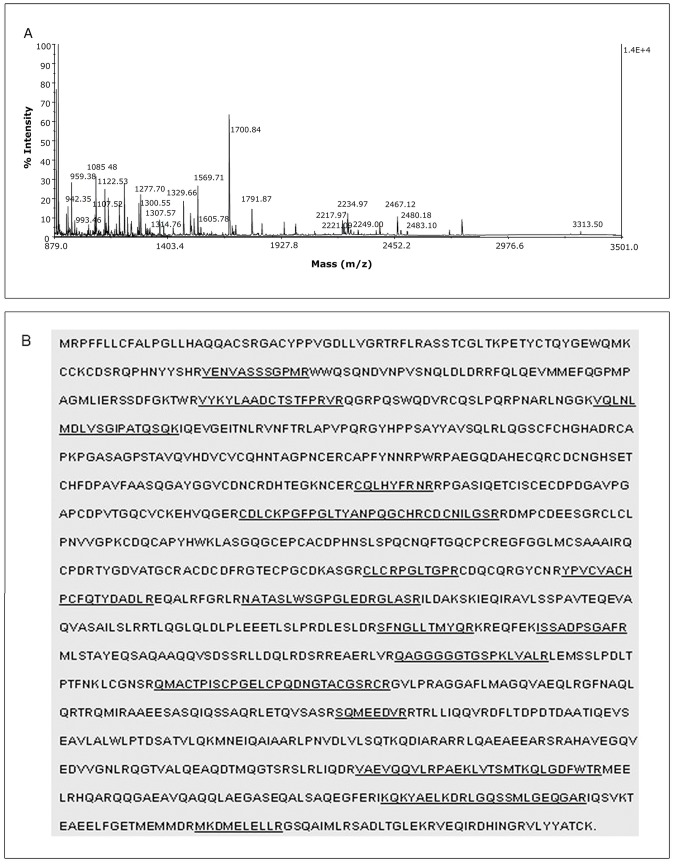
Matching of the amino acid sequences of the 130 kDa protein of *Oncopeltus fasciatus* salivary glands with the human laminin-5 β3 chain amino acid sequence. (**A**). Protein spots cut from the gel were treated with porcine trypsin and the peptides were spotted onto a MALDI-TOF sample plate (Voyager- DE, Applied Biosystem, CA, USA). Peptide mass fingerprints were analyzed using Protein Prospector MS-Fit interface (http://prospector.ucsf.edu). (**B**). Underlined letters represent the amino acid sequences of the salivary gland protein that matched the mass spectrometry data to protein sequences in the NCBI database. The underlined sequences indicate the peptides of the salivary gland protein that were matched with the amino acid sequence of the precursor of the human laminin-5 β3 chain.

One of the protein spots shown in Fig. 4A, that reacted to the biotinylated live parasites (Fig. 4B) was analyzed by mass spectrometry. The analysis of the tryptic digest of this protein spot (Fig. 5A) matched 16 peptides that corresponded to 23% of the human laminin-5 β3 chain precursor sequence (Fig. 5B, underlined sequences).

### Search for laminin subunits in *O. fasciatus* embryo trancriptome

**Figure 6:**
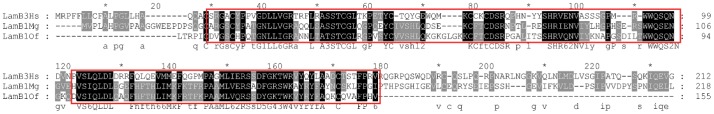
Comparison of laminin subunit β through pairwise alignment of β3 laminin of *Homo sapiens* (LamB3Hs, gi|119613854), β1-like of the turkey *Meleagris gallopavo* (LamB1Mg, gi|326911240) and β1-like of *Oncopeltus fasciatus* (LamB1Of). The alignment of β3 laminin of *H. sapiens* and β1-like of *M. gallopavo* with the partial sequence of β1-like identified in the transcriptome of *O. fasciatus* embryo [28] shows a highly conserved region at the domain VI among these molecules. Black shaded residues are identical or similar amino acids present in all three sequences. Grey shaded residues are identical or similar amino acids present in two of the three sequences. The consensus sequence is represented under alignment lines. In the red rectangles are highlighted the regions with higher similarity between all three sequences. The alignments were performed using GENEDOC software [90].

**Figure 7:**
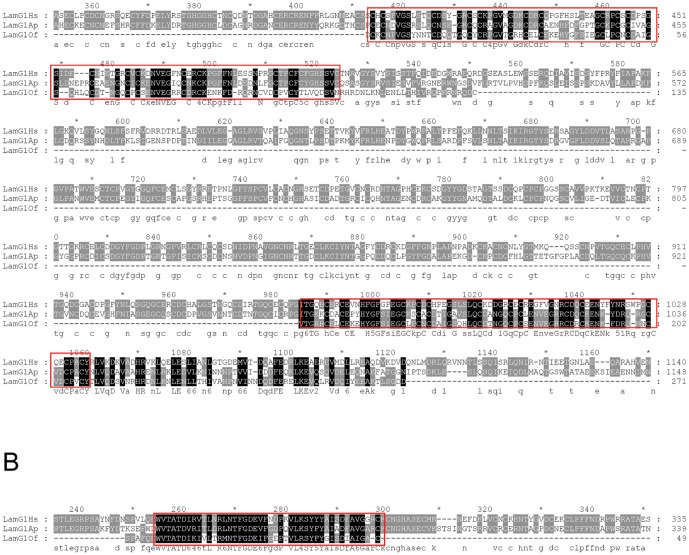
Comparison of laminin subunit γ through pairwise alignment of γ1 of *Homo sapiens* (LamG1Hs, gi|145309326), γ1-like of the hemipteran *Acyrtosiphon pisum* (LamG1Ap, gi|328717115) and γ1-like of *O. fasciatus* (LamG1Of). (**A**) The alignment shows two regions with highly conserved amino acids, consistent with the patterns found in EGF-like domains of γ1 subunits (highlighted in the red rectangles). (**B**) The alignment of the three γ1 subunits also reveals a sequence similar to the domain VI of γ1 of *O. fasciatus* transcriptome [28]. The region similar to domain VI is highlighted in the red rectangles in the alignment. Both in (**A**) and (**B**) black shaded residues are identical or similar amino acids present in all three sequences. Grey shaded residues are identical or similar amino acids present in two of the three sequences. The consensus sequence is represented under alignment lines. The alignments were performed using GENEDOC software [90].

A BLAST analysis applied against the transcriptome of *O. fasciatus* embryo [28] found a β1-like protein showing 51% similarity to human laminin β3 subunit and 65% similarity to *Meleagris gallopavo* laminin β1 subunit (Fig. 6). In addition, two conserved regions, one with 70% similarity to *Acyrtosiphon pisum* domains III and V of laminin γ1 subunit (Fig. 7A) and another one with 77% similarity to a region of the domain VI (N-terminal) of *A. pisum* laminin γ1 subunit are evident (Fig. 7B).

### Immunoblotting assay

**Figure 8:**
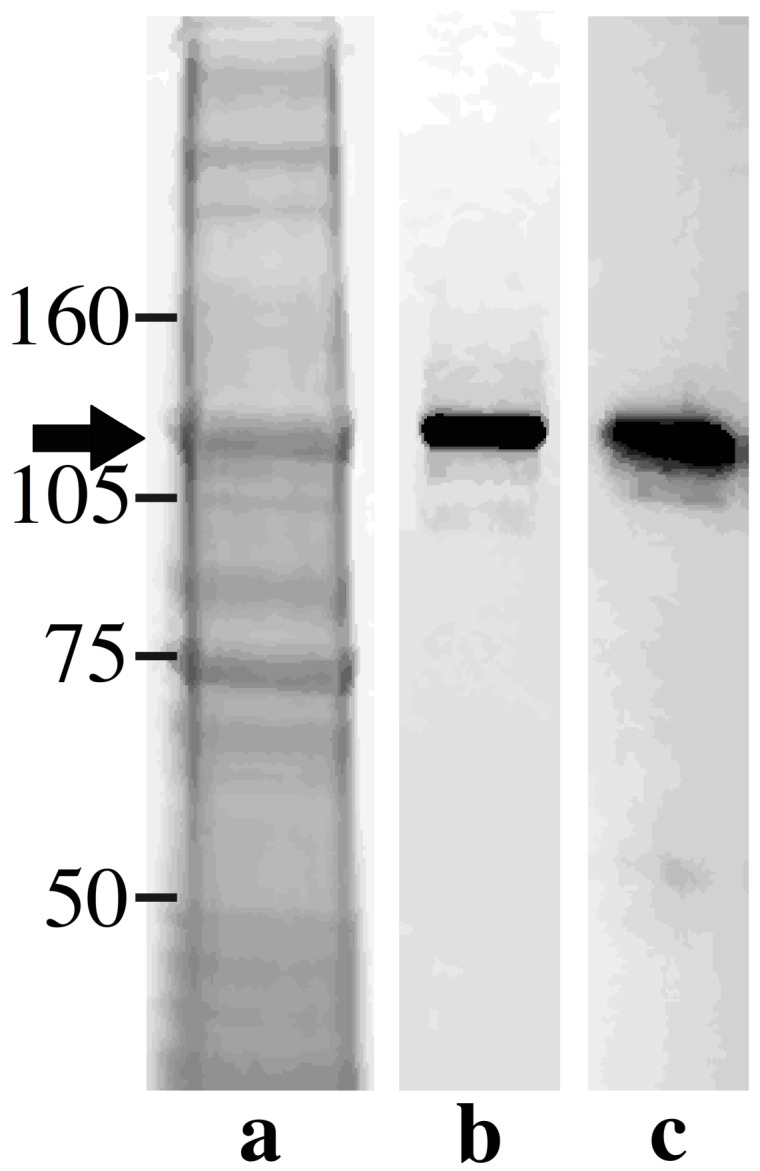
Immunoblotting of the 130 kDa salivary gland protein probed with anti-human laminin-5 β3 chain antibodies. Total salivary gland proteins were extracted and separated by 10% SDS-PAGE (**a**). The 130 kDa band was cut and purified from the gel and the purity of the product was evaluated by 10% SDS-PAGE stained with silver nitrate (**b**). The purified 130 kDa protein was probed with anti-human laminin-5 β3 chain antibodies (**c**). The arrow shows the position of the 130 kDa protein on SDS-PAGE and the stained band by immunoblotting.

Total protein extract and purified p130 protein were separated by SDS-PAGE and stained with silver nitrate (Fig. 8, lanes a and b, respectively). The arrow indicates the p130 protein among all other proteins of the total protein extract (Fig. 8a). Polyclonal antibodies raised against human laminin-5 β3 chain recognized the purified p130 from *O. fasciatus* salivary glands by immunoblotting (Fig. 8, lane c).

### Inhibition of *P. serpens* interaction with *O. fasciatus* salivary glands

**Figure 9:**
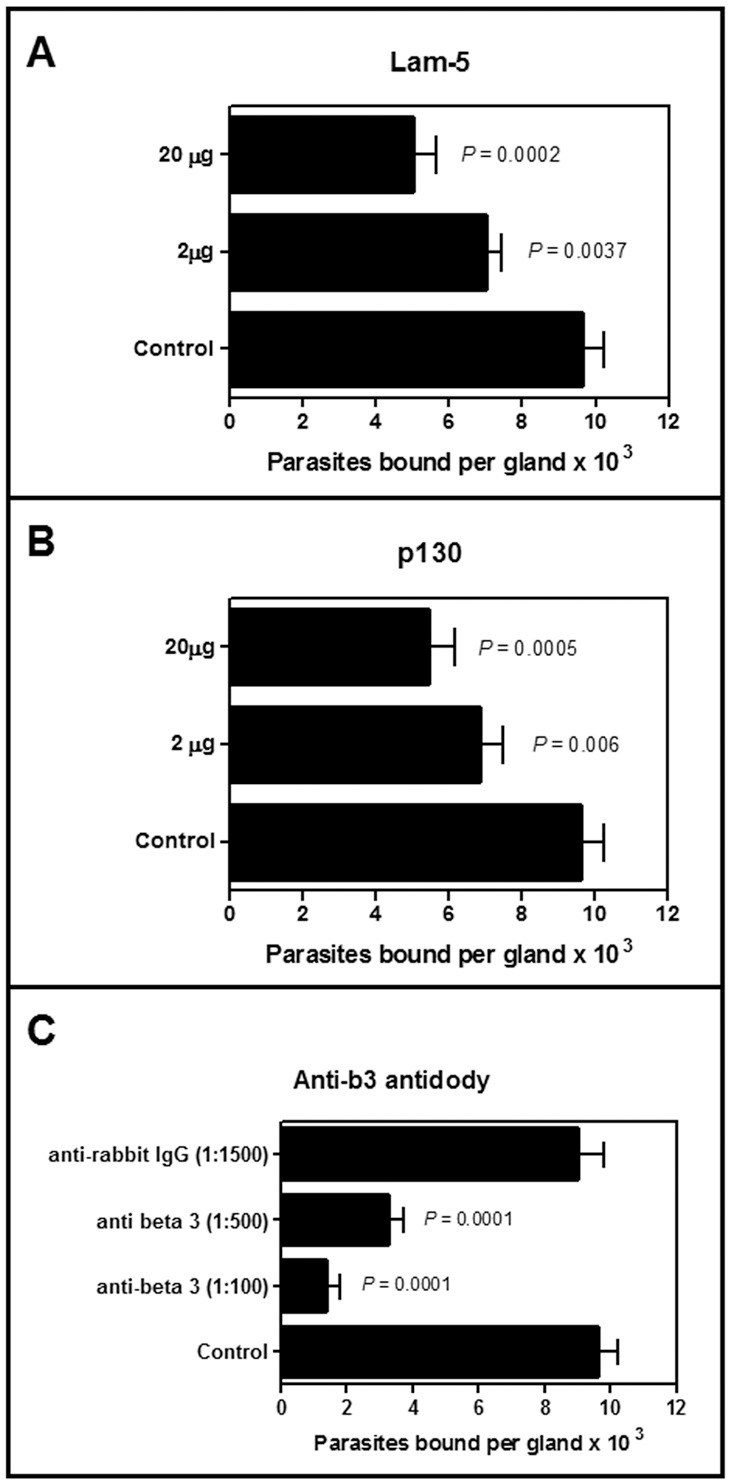
Inhibition of the *in vitro* interaction between *Phytomonas serpens* and salivary glands explanted from *Oncopeltus fasciatus*. The parasites were pre-incubated for 30 min in the presence of human laminin-5 (Lam-5) (**A**) or in the presence of the purified 130 kDa salivary gland protein (p130) (**B**). In a parallel system, the salivary glands were pre-incubated in the presence of anti-human laminin-5 β3 chain antibodies (anti-β3 antibodies) (**C**). In the control systems, the parasites and salivary glands were pre-incubated in the absence of the proteins and antibodies, respectively. The proteins and antibodies were used at the indicated concentrations or dilutions. Each bar represents the mean ± standard error of at least three independent experiments. The *P* values are indicated on the panels.

Control parasite and parasites pre-treated with 2 or 20 µg/ml human laminin-5 or purified p130 obtained from salivary glands were allowed to interact with *O. fasciatus* salivary glands. At the lowest concentration tested, the human laminin-5 and p130 inhibited the binding of the parasites by 27 and 26%, respectively. At the highest concentration tested, the binding of the parasites was inhibited by 48% and 55%, respectively (Fig. 9A and B). Similarly, when the salivary glands were pre-treated with anti-human-5 β3 antibodies at 1∶500 and 1∶100 dilutions before incubation with the parasites, the binding of the parasites was inhibited by 66 and 86%, respectively (Fig. 9C).

## Discussion

Protozoan parasites transmitted by insects cause many diseases in animals (including humans) and plants. Altogether, about 500 million people are infected with *Plasmodium* species (malaria), *Trypanosoma brucei* complex species (African sleeping sickness), *Trypanosoma cruzi* (Chagas disease) and *Leishmania* species (leishmaniasis) [4], [29]. *Phytomonas* species are important plant parasites that cause major economic losses especially in Latin America [1].

The arthropod saliva plays an important role in predatory, hematophagous and phytophagous insects. The saliva is injected into the animal or plant and contains compounds that are able to paralyze the prey or digest their tissues; these compounds may also prevent inflammation and hemostasis of the vertebrate host or interfere with the plant defense mechanisms [30]. The molecular composition of the saliva of a variety of arthropods has been analyzed in detail [31]. In contrast, the surface molecules of the salivary glands that are required for the entry of some important pathogenic parasites and the cell biological events occurring during invasion of the glands are mostly unknown [32]. For example, it is established that the development of the malaria parasite in the mosquito is completed when sporozoites cross the salivary gland epithelium [33]. On the other hand, the mechanism by which the parasite crosses the gland epithelia remains largely undefined; however, it is probably mediated by receptor [34]. Recently, it has been demonstrated that a crucial event for the attachment of *T. rangeli* to the salivary glands of *R. prolixus* is the dephosphorylation of structural phosphotyrosine (P-Tyr) residues at the surface of the glands, which is mediated by a *T. rangeli* P-Tyr ecto-phosphatase [35]. Likewise, *Phytomonas* species need to bind to the external surface of the insect vector salivary glands in order to invade the organ; subsequently, the parasites are transmitted via saliva when the infected insect feeds on another plant [5], [36].

In this study, we examined the interaction between *P. serpens* and the external face of *O. fasciatus* salivary glands, both *in vitro* and *in vivo*. Using scanning electron microscopy we observed that the binding of *P. serpens* to the salivary gland basal lamina occurred through both the flagellum and cellular body. In the trypanosomatid-insect interactions, the adhesion to host tissue seems to occur mainly through the flagella [37]–[40], and binding through the cellular body is rarely observed [41], [42]. In contrast with a previous study that showed *Trypanosoma rangeli* invading *Rhodnius prolixus* salivary glands with the flagellum foremost [27], the passage of *P. serpens* parasites through the basal lamina of *O. fasciatus* salivary gland occurred through the cellular body. In addition, the invasion of several parasites in an area with altered morphology and the presence of suggestive lesions were observed. Punctual damages to the basal lamina were also shown during the penetration of *T. rangeli* into *R. prolixus* and *R. domesticus* salivary glands [43], [44]. Given that proteases that are secreted or present on the surface of many protozoan parasites are involved in tissue invasion [45]–[50], we suggest that the altered morphology in the basal lamina of the *O. fasciatus* salivary glands was promoted by surface and/or secreted protease activities of *P. serpens*, which would aid in gland invasion by the parasites. In fact, our group has consistent evidence of the participation of *P. serpens* proteases in the interaction between this parasite and the salivary glands of *O. fasciatus*. The pre-treatment of *P. serpens* with antibodies raised against the metalloprotease gp63 significantly inhibited the interaction of these parasites with *O. fasciatus* salivary glands [51], [52]. The involvement of a cruzipain-like protease of *P. serpens* in the interaction with *O. fasciatus* salivary glands was also investigated. When the parasites were pre-treated with either protease inhibitors or anti-cruzipain antibodies, a drastic inhibition of binding was observed [53]. Furthermore, the cysteine protease produced by *P. serpens* cleaved at least one polypeptide located at the surface of *O. fasciatus* salivary glands [54].

Intriguingly, when *P. serpens* interacted with *O. fasciatus in vivo*, the parasites were preferentially attached to the regions between the salivary gland lobes, not to the exposed surface of the glands. Considering that *P. serpens* flagellates travel through the hemolymph to reach the insect salivary gland, it is possible that hemolymph molecules may trigger changes on the cell surface of *P. serpens*, enabling the parasites to bind specifically to the regions between lobes. Indeed, in the interaction between *T. rangeli* and *R. prolixus*, a hemolymph factor and/or the distribution of carbohydrate moieties on the salivary glands of *R. prolixus* are considered crucial for the insect vector-parasite interactions [55].

*O. fasciatus* is an emerging model organism, which lacks a sequenced genome [28]. In order to determine putative targets for *P. serpens* binding to the external face of *O. fasciatus* salivary glands, we used a ligand blotting assay developed by our group [56]. These experiments showed that only *O. fasciatus* salivary gland p130 was recognized by the biotinylated live parasites. Proteomic analysis showed a sequence similarity between p130 and the human laminin-5 β3 chain, which was corroborated by the recognition of the purified p130 by antibodies generated against human laminin-5 β3 chain. These similarities and the role of p130 in parasite binding were confirmed by binding inhibition experiments. The inhibition of parasite-gland interaction was dose-dependent when the parasites were pre-treated with human laminin-5 or p130. In addition, the same profile of inhibition was observed when the glands were pre-treated with antibodies raised against human laminin-5 β3 chain.

The latter set of results together with the scanning electron microscopy observations allowed us to assume that p130 is located at the basal lamina of *O. fasciatus* salivary glands. The basal lamina is basically composed of proteins, including laminins [14], [57], [58]. The β1 and β2 chains of the *Drosophila* laminin have been sequenced and these polypeptides are very similar to their vertebrate counterparts. The *Drosophila* β2 chain is 40 and 41% identical to the human and mouse β2 chains, respectively, and 29, 30, and 29% identical to the *Drosophila*, human, and mouse β1 chains, respectively [12], [59]. Intriguingly, the *O. fasciatus* salivary glands transcriptome did not reveal any laminin or laminin-related sequences [60]. At least two explanations can be proposed for these observations. One possibility is that laminin can be synthesized in a tissue or organ and transported to other sites in the body [61]. Another possibility is that because the transcriptome presents a set of mRNA transcribed at the time of its extraction from a given tissue, the laminin-5 β3 chain may not have been transcribed at that time [60]. In contrast, the ovarian and early embryonic transcriptome of *O. fasciatus* has been published and putative laminin genes were found in the sequence data submitted to GenBank [28]. Laminin β1 subunit has previously been described in *Drosophila melanogaster* [59], and both α and γ laminin subunits have been identified in the hemiptera *A. pisum* [62]. We applied a protein BLAST analysis against *R. prolixus* recently sequenced genome, using *H. sapiens* β3 subunit and *A. pisum* γ1 subunit sequences as queries and found predicted protein coding sequences with high similarity to both queries (data not shown). The predicted sequences contain characteristic laminin domains, namely domain VI and epithelium growth factor-like (EGF-like) domains of laminin β3 subunit, as well as laminin β domain and EGF-like domains of laminin γ1, which suggests that such proteins may be present in the basal lamina of the hemiptera *R. prolixus*. The pairwise alignment of *A. pisum* and *H. sapiens* laminin γ1 subunits with protein sequences of the *O. fasciatus* transcriptome also suggests that *O. fasciatus* presents a laminin γ-like subunit. The protein sequences of the *O. fasciatus* putative laminin subunits presented conserved domains, such as the EGF-like in the γ-like subunit, and domain VI of β1 subunit. The domain VI is directly involved with laminin-collagen interactions [63] and the EGF domain has been characterized through the cysteine residues pattern, which is essential for the double stranded beta-sheet conformation [63]. This domain seems to have an essential role in cell attachment and receptor binding [64], [65], corroborating with our hypothesis that p130 is the ligand for a parasite receptor.

Completion of the *Phytomonas* spp life cycle in the insect involves passing through the midgut wall to reach the insect's hemolymph, which predominantly acts as a transport fluid to the salivary glands [5]–[7], [36]. The hemolymph bathes all other insect organs besides the salivary glands, so the presence of specific surface receptors at the external face of salivary glands could be considered a target for *Phytomonas* species, which ultimately attach to and invade that organ [6], [7]. Correspondingly, malaria parasites locate mosquito salivary glands by chemotaxis, suggesting the possibility that chemical component(s) can be identified and synthesized to block or suppress mosquito salivary gland invasion as a malaria transmission blocking strategy [66]. It is noteworthy to mention that laminin-binding proteins have been found on the surface of a variety of pathogens, including the protozoan parasites *L. donovani* [21], [67], [68], *T. cruzi* [19], [69], *Plasmodium* [16], [24], [70], and *Trichomonas* [71], [72]; fungi, such as *Candida albicans* [73]–[75], *Histoplasma capsulatum* [76], and *Paracoccidioides* [77], as well as bacteria, like *Staphylococcus* [78], *Streptococcus* [78], [79], and *Mycobacterium leprae* [80], [81]. The wide array of pathogens that use laminin as their receptor suggests that this strategy has a yet unidentified role that seems to be evolutionarily conserved [82].

The preset study is the first demonstration that a laminin-like molecule from the salivary gland of *O. fasciatus* acts as a receptor for *Phytomonas* binding. The results presented in this investigation are important findings that will support further studies that aim at developing new approaches to prevent the transmission of *Phytomonas* species from insects to plants and vice-versa.

## Materials and Methods

### Insect colony

A milkweed bug (*Oncopeltus fasciatus*) culture kit was purchased from Carolina Biological Supply Company, Burlington, North Carolina, USA. These insects originated the colony we maintain in our laboratory in plastic pitchers under a 12 h light/dark cycle at 28°C with 70–80% relative humidity. The insects were fed with commercially available peeled sunflower seeds and fresh water *ad libitum*. Only adults were used in all the experiments [83]. No field studies were performed in the present work. No specific permits were required for the described studies.

### Parasites

*Phytomonas serpens* parasites (isolate 9T, CT-IOC-189), isolated from tomato (*Lycopersicon esculentum*), was provided by Dr. Maria A. de Sousa, Trypanosomatid Collection, Instituto Oswaldo Cruz, Rio de Janeiro, Brazil. The parasites were grown in Warren medium (37 g/l brain-heart infusion, 1 mg/l folic acid, 10 mg/l hemin) supplemented with 10% fetal calf serum at 26°C. Parasites were harvested at early stationary growth phase by centrifugation (10 min at 2,000× *g*) and washed three times in TBS. Cellular viability was assessed by motility before and after all procedures. The viability of the parasites was never affected by the conditions used in this study.

### *Ex vivo* interaction of *Phytomonas serpens* with *Oncopeltus fasciatus* salivary glands

Salivary glands were carefully dissected and explanted from adult insects seven days after moulting. The glands were placed in a Petri dish containing TBS (150 mM NaCl, 10 mM Tris, pH 7.2) at 4°C. Ten pairs of explanted salivary glands were incubated in a suspension containing 10^7^ parasites in 100 µl TBS, supplemented with 1% bovine serum albumin. After an incubation period of 60 min at 26°C, unbound parasites were removed by three consecutive washes with TBS.

### *In vivo* experimental infection of *Oncopeltus fasciatus* with *Phytomonas serpens*

Parasites were grown as described above, harvested at early stationary growth phase by centrifugation (15 min at 200× *g*) and washed three times in sterile PBS (150 mM NaCl, 20 mM sodium phosphate, pH 7.2), at 4°C. The flagellates were then resuspended in sterile PBS (pH 7.2), and 4 µl of this suspension (5×10^4^ cells parasites) were injected in each insect (adult insects seven days after moulting). The parasites were injected laterally into the thorax, between the second and third thoracic segments of the insects, using a 10-µl Hamilton syringe [84]. Control insects were injected with 4 µl sterile PBS. The salivary glands of the insects were dissected and explanted at 2, 6, 24, 48 and 72 hours post-infection [27].

### Scanning electron microscopy of salivary glands

The salivary glands obtained from both *in vitro* and *in vivo* experimental infections were washed three times with TBS and fixed in a solution containing 2.5% (v/v) glutaraldehyde, 4% (w/v) freshly made formaldehyde, 3.7% (w/v) sucrose, and 5 mM CaCl_2_ in a 0.1 M cacodylate buffer, pH 7.2, for 1 h at 26°C. After fixation, the glands were post-fixed in 1% (v/v) osmium tetroxide, 0.8 M potassium ferricyanide, and 5 mM CaCl_2_ in 0.1 M cacodylate buffer, pH 7.2, for 1 h at 26°C. The glands were dehydrated in ethanol, dried using the CO_2_ critical point method [85] in a Balzers apparatus model CDP-20, mounted on aluminum stubs with double coated carbon conductive tape, and sputtered with gold in a Balzers apparatus model FC-9646. Scanning electron microscopy observations were made under a Jeol JSM-5310 electron microscope.

### Sodium dodecyl sulfate-polyacrylamide gel electrophoresis (SDS-PAGE) and gel-membrane protein transfer

Ten pairs of intact glands were frozen in 100 µl TBS containing 0.1% SDS supplemented with a protease inhibitor cocktail (100 µM E-64, 10 µM 1,10-phenanthroline, 10 µM pepstatin A, and 1 mM PMSF). After thawing, the glands were homogenized using a Teflon coated microtissue grinder. Homogenates were centrifuged at 8,000× g for 10 min at 4°C and supernatant aliquots corresponding to 40 µg protein mixed with sample buffer (125 mM Tris, pH 6.8, 4% SDS, 20% glycerol, 0.002% bromophenol blue) were separated by 12% SDS-PAGE at 4°C, 150 V, and 60 mA, using a protein electrophoresis apparatus (Bio-Rad Laboratories, CA, USA). The gels were then stained for 1 h with 0.2% Coomassie brilliant blue R-250 in methanol-acetic acid-water (50∶10∶40) and washed in the same solvent. The molecular mass of the polypeptides was calculated according to the mobility of the “Full Range Rainbow” molecular mass standards (GE Healthcare, NJ, USA). Proteins separated on SDS-PAGE were transferred at 4°C, 100 V, and 300 mA for 2 h in 25 mM Tris-base, 200 mM glycin, and 20% methanol (pH 8.0) from the gels to polyvinylidene difluoride (PVDF) membranes using protein transfer cells (Bio-Rad Laboratories, CA, USA).

### Biotinylation of parasite surface proteins

Suspensions containing live parasites (10^8^ parasites/ml) in PBS, pH 8.0, were treated with 0.1 mg of impermeable biotin (Sulfo-NHS-Lc-Biotin - Pierce Biotechnology, IL, USA) per ml of reaction volume for 20 min at 4°C. The parasites were washed three times in TBS to remove the unbound biotin [56].

### Ligand blotting

The ligand blotting assays were carried out as previously described [56]. Briefly, the PVDF membranes were blocked in solution A (150 mM NaCl, 0.05% Tween 20, 1% bovine serum albumin (BSA), 10 mM Tris, pH 7.2) for 15 h at 4°C, in order to prevent non-specific binding [86], before incubation with live biotinylated parasites (10^8^ cells/ml) for 1 h at 26°C. After the incubation, the membranes were washed three times in solution B (150 mM NaCl, 0.05% Tween 20, 10 mM Tris, pH 7.2), incubated in solution B containing peroxidase-labeled streptoavidin (0.1 µg/ml) for 1 h at 26°C, and then washed three times in solution B. The bands containing live biotinylated parasites were detected with an ECL kit (GE Healthcare, NJ, USA) according to the manufacturer's protocol.

### Two-dimensional (2D) gel electrophoresis

Two-dimensional polyacrylamide gel electrophoresis was performed with a Multiphor II unit (GE Healthcare, NJ, USA) on an immobilized pH gradient 4 to 7 (Immobiline DryStrip, 7 cm, pH 4–7, GE Healthcare, NJ, USA) for the first dimension and SDS-PAGE on a 10% linear mini-gel (Bio-Rad system) for the second dimension. Samples were prepared by suspending ten pairs of salivary glands in lysis buffer (8.99 M urea, 0.02% Triton X-100, 0.13 M DTT, 0.02% (v/v) Pharmalyte 3–10, and 8 mM PMSF) followed by incubation at room temperature for 30 min and centrifugation at 8,000× *g* for 10 min at 4°C. A volume of the supernatant containing 200 µg protein was mixed with a solution containing urea in order to achieve rehydration solution concentrations and then loaded onto the strip. Proteins were focused according to the manufacturer's instructions (GE Healthcare, NJ, USA). The gel strip was then loaded onto the polyacrylamide-SDS vertical gel and the proteins were separated as previously described [87]. Gels were then stained with Coomassie Blue G-250 [88], scanned with an Image Scanner (GE Healthcare, NJ, USA), and analyzed with the Image Master 2D Platinum software (GE Healthcare, NJ, USA). The isoelectric point values of the proteins of interest were determined using a linear 4–7 distribution, and the relative molecular mass (Mr) was determined based on protein low Mr markers (GE Healthcare, NJ, USA).

### Peptide mass fingerprinting

Protein spots cut from 2D gels were destained with 25 mM NH_4_HCO_3_ in 50% acetonitrile (ACN) and treated with porcine trypsin (Promega, WI, USA) as previously described [89]. Peptides were extracted with 50% ACN and 5% trifluoroacetic acid (TFA), and the resulting solution was dried in a Speed Vac (GE Biosciences, NJ, USA) to reduce the volume to 10 µl. One µl of the peptide solution was mixed with 1 µl of a saturated solution of α-cyano-4-hydroxycinnamic acid matrix in 50% ACN and 1% TFA. The mixture was spotted onto a MALDI-TOF sample plate (Voyager-DE, Applied Biosystem, CA, USA). Trypsin autolysis peptides masses 842.5 and 2211.1 and calibration mixture 2 (Sequazyme Peptide Mass Standard kit, PerSeptive Biosystems, CA, USA) were used as internal and external standards, respectively. Spectra were obtained in reflectron-delayed extraction mode with high resolution for 800–4000 Da range. Peptide mass fingerprints were analyzed using Protein Prospector MS-Fit interface (http://prospector.ucsf.edu) that matched the mass spectrometry data to protein sequences in the NCBI database. The criteria for identification were first a MOWSE score above 10^4^, at least a 100-fold difference in MOWSE score from the second possible hit, 20% of protein cover, and 8 matched peptides.

### Search for laminin subunits in the *O. fasciatus* embryo transcriptome

Basic local alignment search tool (BLAST) was used for comparing the amino-acid sequences of the transcriptome of *O. fasciatus* embryo [27] with human laminin β3 subunit, as well as with the turkey *Meleagris gallopavo* laminin β1 subunit and the hemipteran *Acyrtosiphon pisum* laminin γ1 subunit. The pairwise alignments were performed using GENEDOC software [90].

### Purification of the laminin-like protein from the salivary glands

Proteins from five hundred pairs of salivary glands (25 mg total protein) were extracted and separated by 10% SDS-PAGE as described above. The 130 kDa band was visualized after incubation of the gel in a 1 M potassium chloride solution. The band was then cut from the gel and incubated in the elution solution (50 mM sodium bicarbonate, 0.1% SDS, pH 7.8) for 1 h at 37°C. After incubation, the preparation was centrifuged at 8,000× g for 20 min and the proteins were precipitated from the supernatant with 80% acetone at −20°C. The purity of the product was evaluated by SDS-PAGE stained with silver nitrate, as previously described [91].

### Immunoblotting assay

PVDF membranes containing salivary gland proteins were blocked in solution A for 15 h at 4°C and then incubated with goat polyclonal antibodies raised against the human-5 β3 laminin chain (sc-7651, Santa Cruz Biotechnology) at a 1∶500 dilution. The secondary antibody used was peroxidase-conjugated rabbit anti-goat IgG (A 4174, Sigma-Aldrich) at 1∶10,000 dilution. The antibody dilutions and membrane washes were performed in solution A. Bound antibodies were then detected with an ECL kit (GE Biosciences, NJ, USA) according to the manufacturer's protocol.

### Inhibition of the *ex vivo* parasite-salivary gland interaction

Ten pairs of explanted salivary glands were incubated for 60 min at 26°C in 100 µl TBS that contained 10^7^ parasites. These flagellates had been pre-treated for 30 min at 26°C in the absence (control) or in the presence of 2 or 20 µg/ml human laminin-5 or the purified p130. Alternatively, before the interaction ten pairs of salivary glands were maintained for 30 min at 26°C in the absence (control) or in the presence of anti-human-5 β3 laminin chain goat IgG antibodies at 1∶500 dilution or anti-rabbit IgG at 1∶100 dilution (negative control). After incubation, unbound parasites were removed by three consecutive washes in TBS and the number of bound parasites was determined as previously described [92].

### Statistical analysis

The experiments of inhibition of the *in vitro* interaction between *P. serpens* and salivary glands explanted from *O. fasciatus* were performed in triplicates. The results are presented as the mean and standard error of the mean (SEM). Normalized data were analyzed by one-way analysis of variance (ANOVA) and differences between groups were assessed using the Student Newman-Keuls post-test. A *P* value of <0.05 was considered significant.
